# Topographical Analysis of the Subependymal Zone Neurogenic Niche

**DOI:** 10.1371/journal.pone.0038647

**Published:** 2012-06-20

**Authors:** Ana Mendanha Falcão, Joana Almeida Palha, Ana Catarina Ferreira, Fernanda Marques, Nuno Sousa, João Carlos Sousa

**Affiliations:** 1 Life and Health Sciences Research Institute (ICVS), School of Health Sciences, University of Minho, Braga, Portugal; 2 ICVS/3B’s - PT Government Associate Laboratory, Guimarães, Braga, Portugal; Instituto de Medicina Molecular, Portugal

## Abstract

The emerging model for the adult subependymal zone (SEZ) cell population indicates that neuronal diversity is not generated from a uniform pool of stem cells but rather from diverse and spatially confined stem cell populations. Hence, when analysing SEZ proliferation, the topography along the anterior-posterior and dorsal-ventral axes must be taken into account. However, to date, no studies have assessed SEZ proliferation according to topographical specificities and, additionally, SEZ studies in animal models of neurological/psychiatric disorders often fail to clearly specify the SEZ coordinates. This may render difficult the comparison between studies and yield contradictory results. More so, by focusing in a single spatial dimension of the SEZ, relevant findings might pass unnoticed. In this study we characterized the neural stem cell/progenitor population and its proliferation rates throughout the rat SEZ anterior-posterior and dorsal-ventral axes. We found that SEZ proliferation decreases along the anterior-posterior axis and that proliferative rates vary considerably according to the position in the dorsal-ventral axis. These were associated with relevant gradients in the neuroblasts and in the neural stem cell populations throughout the dorsal-ventral axis. In addition, we observed spatially dependent differences in BrdU/Ki67 ratios that suggest a high variability in the proliferation rate and cell cycle length throughout the SEZ; in accordance, estimation of the cell cycle length of the neuroblasts revealed shorter cell cycles at the dorsolateral SEZ. These findings highlight the need to establish standardized procedures of SEZ analysis. Herein we propose an anatomical division of the SEZ that should be considered in future studies addressing proliferation in this neural stem cell niche.

## Introduction

The subependymal zone (SEZ), generally described as a thin layer of proliferative cells lining the lateral wall of the lateral brain ventricles, is a major source of multipotent neural stem cells (NSCs) in the adult brain [1], [2]. The fate of this pool of stem cells is to generate new neurons that migrate anteriorly along the rostral migratory stream (RMS) towards the olfactory bulb where they differentiate into different types of interneurons [3], [4]. Additionally, it was shown that SEZ NSCs generate oligodendrocytes [5], [6]. Alterations in the proliferative and migratory profile of the SEZ NSC population are extensively described for several animal models of neurological disorders, such as Alzheimer’s and Parkinson’s diseases, stroke and epilepsy [7]. Altogether, such studies have raised expectations for the development of endogenous regenerative therapies based on the manipulation of the SEZ neurogenic niche. However, to fully explore the regenerative potential of the SEZ stem cell niche, a better knowledge of how the niche is maintained and regulated, both in physiological and pathological conditions, is needed.

Recent studies demonstrated that, in mice, the SEZ stem cell niche is not topographically and functionally uniform; indeed, the SEZ niche is not restricted to the lateral walls of the ventricles, but rather extends to more dorsal portions of the ventricle walls [8] and to the RMS [9]. In accordance, several reports extend the analysis of the SEZ to the beginning of the RMS [10]–[13]. In addition, it is becoming increasingly evident that the SEZ NSC population is heterogeneous as supported by *in vitro* studies which show a large variation in the number of neurosphere forming cells extracted from serial brain slices along the anterior-posterior axis [14]. Furthermore, there is also evidence that the expression of transcription factors by NSCs varies according to their position along the ventricular neuraxis [15]–[17]. Interestingly, a correlation between the regionalization of type B cells and cell-fate specification has also been described [18]; for example, SEZ cells were found to generate not only GABAergic neurons, but also glutamatergic olfactory bulb interneurons specifically derived from the dorsal SEZ [8].

Taken together, the literature reflects the heterogeneity and complexity of the SEZ stem cell niche and anticipates the pitfalls that may occur when data obtained from specific regions in the anterior-posterior and dorsal-ventral axes are used for extrapolations to the entire SEZ. Also of consideration, the lack of consistency or specificity in topographical mapping may generate discrepancies between studies and mask relevant changes in specific regions when the analysis is made as a whole [19]. Therefore, we thought of relevance to characterize the proliferation pattern of SEZ cells throughout the anterior-posterior and dorsal-ventral axes. Taking into consideration the profile encountered, we propose a standard division for the anterior-posterior SEZ and define the dorsal-ventral regions in the SEZ based on differences in cell proliferation and on anatomic parameters.

## Results

### Analysis of Cell Proliferation Rate Along the Anterior-posterior Axis

**Figure 1:**
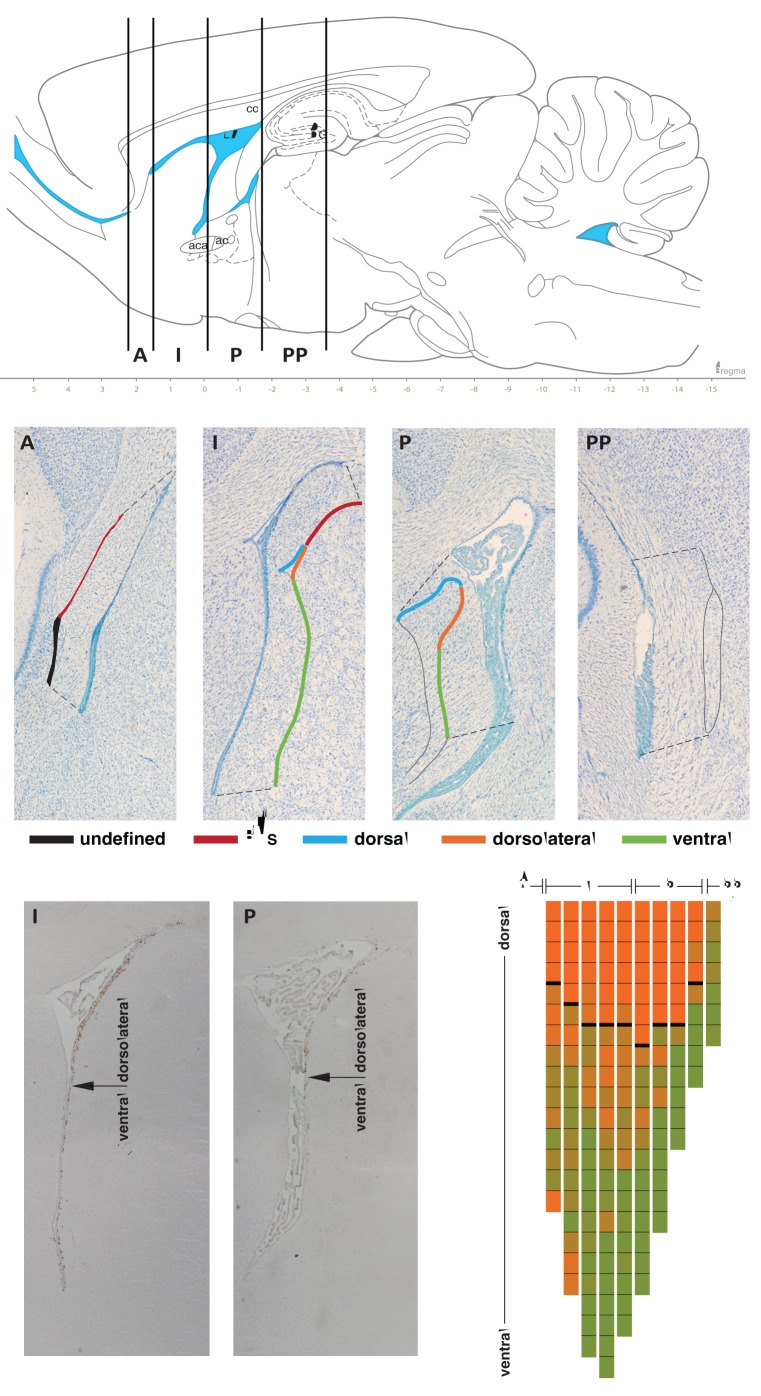
Representation of the subependymal zone divisions defined at the anterior-posterior and dorsal-ventral axes. In the *upper panel* four anterior to posterior divisions are defined according to the SEZ anatomical heterogeneity along the neuraxis: anterior (A), intermediate (I), posterior (P) and post-posterior (PP). For the established divisions, regions are further defined in a dorsal to ventral SEZ orientation, as outlined in the colored traces (*middle panel*): rostral migratory stream (RMS; red trace), dorsal (blue trace), dorsolateral (orange trace), and ventral (green trace). In the anterior division of the SEZ, the area containing proliferating cells that cannot be defined as RMS is designated undefined (black trace). In the post-posterior division of the SEZ, few proliferating cells are found lining the ventricle wall and therefore no dorsal-ventral region is defined (ventricle walls outlined in grey). The topography of each region varies across the SEZ divisions (*middle panels*). Along the lateral wall of the brain ventricles proliferation decreases from the most dorsal portion to the ventral tip (*left lower panels*). Dorsolateral and ventral SEZ regions were defined, by subdividing the lateral wall of the ventricle in 150 µm-long contiguous fragments, and proliferating cells per area along the anterior to posterior axis were counted. The density of proliferating cells is graphically and spatially represented in the colored tiled map (*right lower panel*); the color scale ranges from orange to green, representing higher to lower density of proliferating cells, respectively. A pronounced decrease in the number of proliferating cells is observable at specific locations of the lateral wall defining the boundary between dorsolateral and ventral SEZ (represented by an arrow in the *left lower panels* and by a bold line in each column of the colored map). ac, anterior commissure; aca, anterior commissure, anterior part; cc, corpus callosum; DG, dentate gyrus; LV, lateral ventricle.

**Figure 2:**
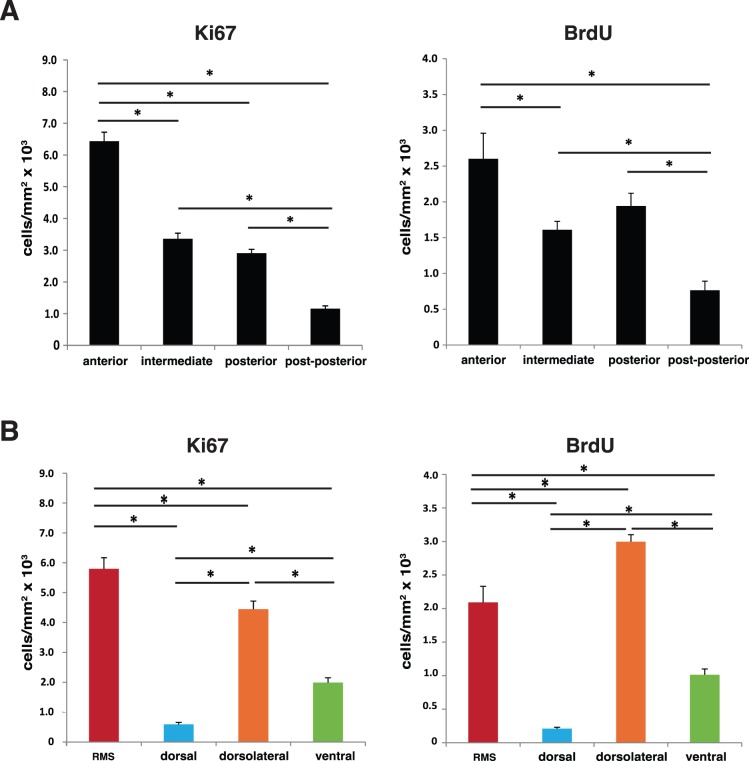
The subependymal zone cell proliferation pattern is dependent on the anterior-posterior and dorsal-ventral axes position. (A) SEZ total proliferation analysis throughout anterior-posterior divisions shows the highest number of Ki67 and BrdU positive cells in the anterior SEZ, decreasing along the intermediate, posterior and post-posterior levels. (B) Cell proliferation varies according to the SEZ dorsal-ventral axis position. Proliferation is expressed as number of Ki67 or BrdU positive cells per area (mm^2^). The threshold value for statistical significance was set at 0.05 (* p<0.05).

Analysis of the SEZ cell proliferation rate along the anterior-posterior axis, as defined in the material and methods section and in Figure 1, revealed that the anterior SEZ displays the highest number of Ki67 positive cells per mm^2^ (6.40±0.27×10^3^) that comparatively decreases 48% and 52% at the intermediate and posterior SEZ divisions, respectively (Figure 2A). Similarly, analysis of proliferation with BrdU revealed that at the intermediate and posterior levels of the SEZ, BrdU incorporation was 45% and 34% lower than in the anterior division (2.86±0.29×10^3^ BrdU positive cells/mm^2^) (Figure 2A). These results showed that the SEZ cell proliferation rate is higher in the anterior division than in the intermediate and posterior divisions and that the latter two display very similar proliferation patterns. The proliferation analysis was extended further posteriorly along the anterior posterior axis, into a division here designated post-posterior which is anatomically found at the same level of the hippocampus, the other major neurogenic niche of the adult brain. Post-posterior SEZ exhibited the lowest proliferation rates of the anterior-posterior axis with both Ki67 (1.15±0.09×10^3^ cells/mm^2^) and BrdU (0.77±0.12×10^3^ cells/mm^2^) markers (Figure 2A). The present data highlights the heterogeneity in cell proliferation rates in the SEZ along the anterior-posterior axis.

### Analysis of Cell Proliferation Rates Along the Dorsal-ventral Axis

Along the dorsal-ventral axis, proliferation was assessed separately in every 150 µm length fragment, beginning at the top of the lateral wall (dorsally positioned) to the ventral tip. The proliferation rate, assessed by Ki67 and BrdU labelling, decreased in the lateral wall along the dorsal to ventral axis (Figure 1, lower panel). Interestingly, as for the anterior-posterior axis, there was a position in the dorsal-ventral axis where the proliferation rate decreased steeply (indicated by the arrow in the lower panel of Figure 1). These observations prompted for the division of the lateral wall of the SEZ in two different regions: dorsolateral and ventral. The dorsolateral SEZ comprises the dorsal part of the lateral wall and extends to the beginning of the ventral SEZ. At this point there is a directional switch of the lateral wall that starts elongating perpendicular to the dorsal SEZ. Thus, taking into account these observations, four distinct regions were considered to estimate the proliferation rates throughout the dorsal-ventral axis: RMS (specifically the beginning of the RMS), dorsal, dorsolateral and ventral (illustrated in Figure 1 middle and lower panels). To the best of our knowledge, this is the first study that separately estimates proliferation rates in different dorsal-ventral regions of the SEZ.

Examination of both Ki67 and BrdU positive cells along the SEZ dorsal-ventral axis revealed major differences in cell proliferation rates between the four defined regions (Figure 2B). The RMS displayed the highest values for Ki67-positive cells (5.80±0.37×10^3^ cells/mm^2^), with this value decreasing 23% in the dorsolateral region (4.45±0.27×10^3^ cells/mm^2^). In contrast, the dorsal SEZ presented a number of Ki67 positive cells/mm^2^ of only approximately 10% comparatively to the RMS and the dorsolateral SEZ, the lowest proliferation densities of the four regions. The ventral SEZ also displayed low values for proliferation, 55% bellow the value displayed by the anatomically contiguous dorsolateral SEZ. Interestingly, the SEZ proliferation pattern estimated by BrdU incorporation did not completely mirror the data obtained for Ki67. The number of BrdU positive cells in the dorsolateral SEZ was significantly higher (p <0.01) than in the RMS (Figure 2B).

### Combined Analysis of Proliferation in the Anterior-posterior and Dorsal-ventral Axes

**Figure 3:**
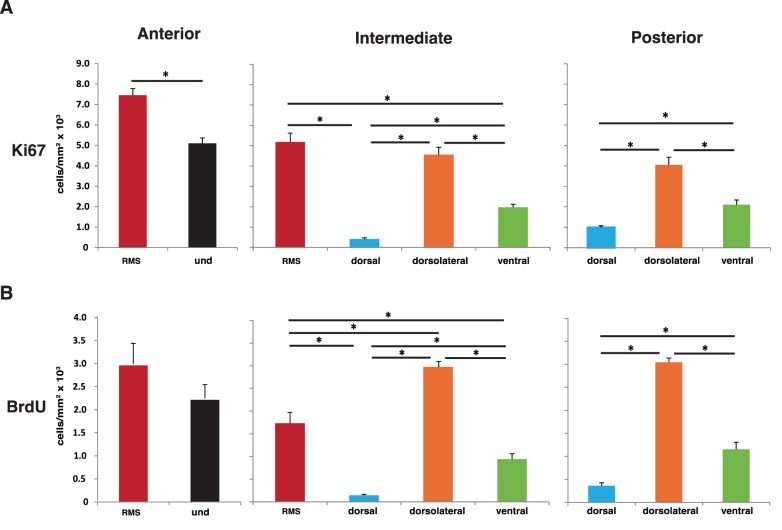
Combinatorial analysis of cell proliferation in the subependymal zone anterior-posterior and dorsal-ventral axes. The proliferation rate in the different dorsal-ventral regions was assessed at the anterior, intermediate and posterior levels either with Ki67 **(A)** or BrdU **(B)**. Proliferation pattern analysis in dorsal-ventral SEZ regions along the defined anterior to posterior axis revealed that proliferation in the RMS significantly decreased from the anterior to the intermediate division. Cell proliferation in the dorsal, dorsolateral and ventral regions was not significantly affected in the intermediate to posterior divisions transition. Proliferation is expressed as number of Ki67 and BrdU positive cells per area (mm^2^). The threshold value for statistical significance was set at 0.05 (* p<0.05).

Since the proliferation rates vary along the anterior-posterior axis, as described above, the four different dorsal-ventral regions were further analysed separately in the anterior, intermediate and posterior divisions (Figure 3). According to the criteria used to define these four regions, only the RMS is identified in the anterior SEZ. The intermediate SEZ comprises all four regions and the posterior SEZ contains the dorsal, dorsolateral and ventral regions. While the proliferation rates of intermediate and posterior SEZ of dorsolateral and ventral regions remained constant, RMS proliferation, assessed both by Ki67 and BrdU, significantly decreased from the anterior to the intermediate divisions. In contrast, proliferation in the dorsal SEZ increased from the intermediate to the posterior division (Figure 3A and 3B).

### Analysis of the Neuroblast and NSC Populations Along the SEZ Axes

**Figure 4:**
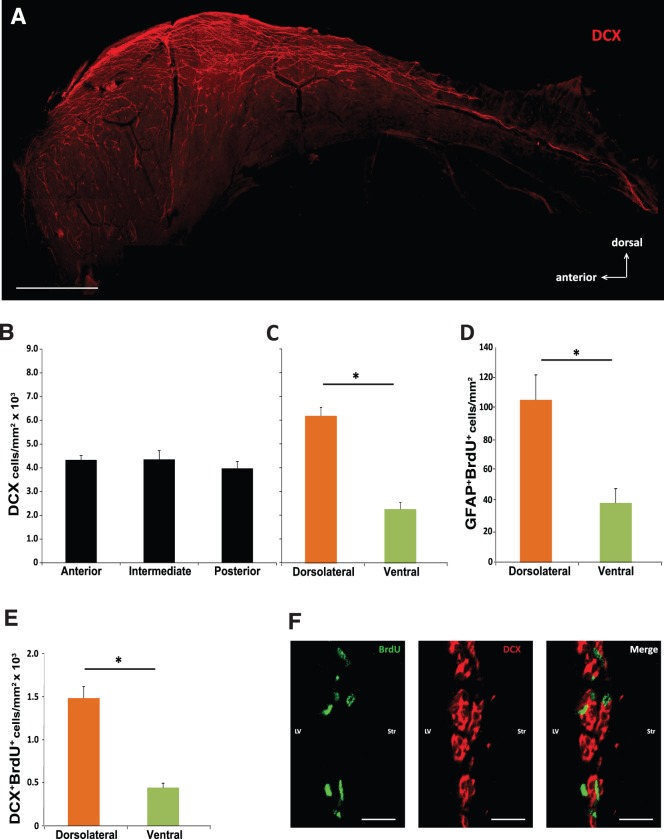
Neural stem and progenitor cells decrease along the subependymal zone dorsal-ventral axis. A DCX wholemount staining for the lateral wall is represented in **(A)** (Scale bar = 1 mm). DCX positive cell rates were estimated through the lateral wall for anterior, intermediate and posterior SEZ **(B)**, dorsolateral and ventral SEZ **(C)**. BrdU retaining cells were double stained with GFAP and assessed in the dorsolateral and ventral SEZ **(D)**. The same analysis was performed for proliferating neuroblasts (double BrdU/DCX positive cells) **(E)**. The images for the BrdU, DCX and BrdU/DCX staining are represented in **(F)** (Scale bar = 20 µm). LV, lateral ventricle; Str, striatum. All results are expressed as number of positive cells per area (in mm^2^). The threshold value for statistical significance was set at 0.05 (* p<0.05).

The observed dissimilarities in the proliferative patterns given by the proliferation markers Ki67 and BrdU led us to discriminate which cell type population/populations could explain these findings. A 2 hours BrdU pulse labels mostly fast dividing cells, i.e., neuroblasts and transit amplifying progenitors (TAPs). In order to obtain a comprehensive view of the SEZ neuroblasts, a wholemount staining of the entire wall of the SEZ was performed. Figure 4A shows a pronounced distribution of the neuroblasts towards the dorsal part of the lateral wall equivalent to the dorsolateral SEZ. Furthermore, the estimation of the rates of neuroblasts (DCX positive cells) in the various regions showed similar rates from the anterior to the posterior SEZ (Figure 4B). Conversely, at the dorsal-ventral axis, the dorsolateral SEZ (6.20±0.35×10^3^ DCX positive cells/mm^2^) displayed higher rates for neuroblasts when compared with the ventral SEZ (2.28±0.27×10^3^ DCX positive cells/mm^2^) (Figure 4C). This finding is in line with the proliferative pattern referred above. Importantly, the analysis of proliferating neuroblasts (double DCX/BrdU positive cells, Figure 4F) provided a similar profile (Figure 4E). Furthermore, BrdU retaining cells double labelled with GFAP (an approach to label NSC) revealed a decreasing gradient from the dorsolateral SEZ to the ventral SEZ (Figure 4D).

### Estimation of the BrdU/Ki67 Ratio throughout the SEZ Axes

**Figure 5:**
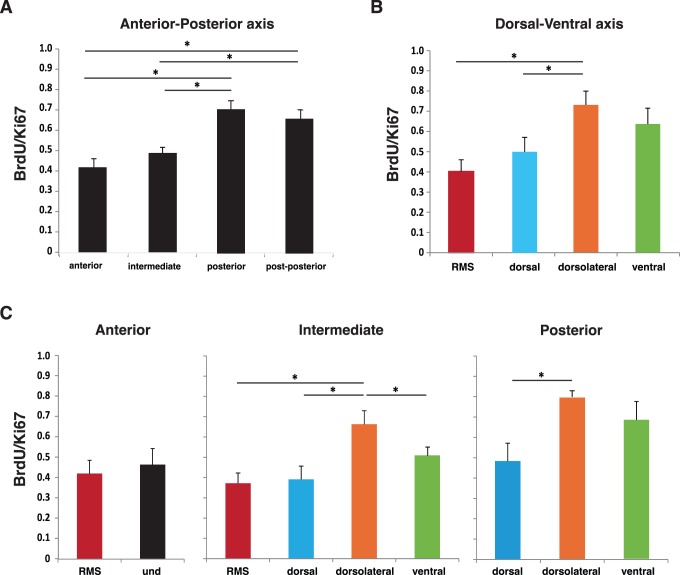
The BrdU/Ki67 ratio differs throughout the subpendymal zone. The SEZ total BrdU/Ki67 ratio is represented for the anterior-posterior **(A)** and dorsal-ventral axes **(B)**. For the different dorsal-ventral regions the BrdU/Ki67 ratios were assessed at the anterior, intermediate and posterior levels **(C)**. The threshold value for statistical significance was set at 0.05 (* p<0.05).

To verify whether the oscillations in proliferation densities along the entire SEZ resulted from diverse mitotic rates, the ratio between BrdU and Ki67 throughout the SEZ was next determined. This ratio provides an estimation of cell cycle length since Ki67 labels all phases of the cell cycle (excluding G0), and BrdU is incorporated exclusively in the S phase [20]. It is important to note that the length of the S phase remains relatively constant whereas the G1 phase regulates cell cycle length [21]. A 2 hours BrdU pulse was given to avoid secondary cell divisions that would allow BrdU dilution; thus the BrdU/Ki67 ratio provides an estimation of the cell cycle length [20], [22]. Interestingly, the posterior and post-posterior SEZ presented the highest BrdU/Ki67 ratio, when compared to anterior and intermediate SEZ (p?0.01)(Figure 5A). Considering the dorsal-ventral axis regionalization, again major differences were found in the BrdU/Ki67 ratio between the dorsolateral and the dorsal SEZ and RMS (40% and 45% decreased, respectively, when compared to the dorsolateral SEZ) (Figure 5B). Combined analysis of BrdU/Ki67 in the anterior-posterior and dorsal-ventral axes revealed similar results; however, the BrdU/Ki67 ratio at the ventral SEZ was lower than at the dorsolateral SEZ at intermediate levels (Figure 5C).

### Estimation of the Neuroblasts Cell Cycle Length throughout the SEZ Axes

**Figure 6:**
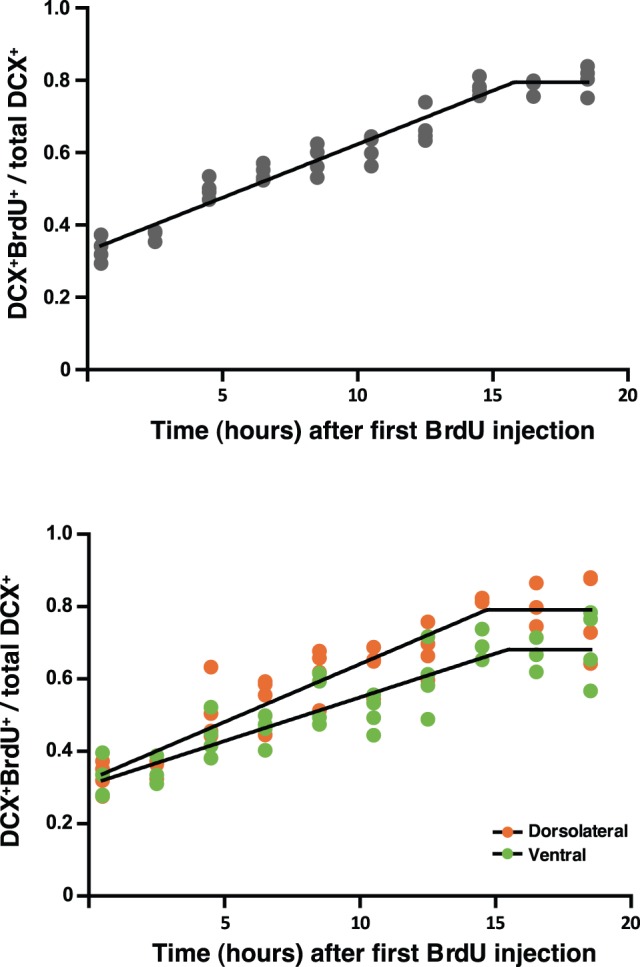
Estimation of the cell cycle length of the DCX positive cell population reveals differences between dorsolateral and ventral subependymal zone at intermediate levels. A cumulative BrdU labeling protocol was performed to determine cell cycle length for DCX positive cells. The time points for BrdU injections are plotted against the percentage of the total DCX population (DCX positive cells) that is proliferating (double DCX/BrdU positive cells) at each time point. When this percentage is constant (the graphic reaches a plateau) it is named Growth Fraction (GF). The parameters to calculate cell cycle length (Tc) are obtained from the following parameters: GF and slope of the first linear fragment. This procedure was performed for DCX positive cells from the entire SEZ (A) or from dorsolateral and ventral SEZ at intermediate levels separately (B).

The cell cycle length estimated for the overall neuroblasts population (labeled by DCX) in the SEZ was of 26.9 (0.23) hours; this value was calculated from the parameters given by the graph of Figure 6A, (GF = 0.79, slope = 0.02957). The same analysis was performed to estimate neuroblasts cell cycle length along the anterior-posterior axis (anterior, intermediate and posterior SEZ) and dorsal-ventral axis (dorsolateral and ventral SEZ). Although no significant differences were found in the neuroblasts cell cycle length along the anterior-posterior axis [anterior, intermediate and posterior levels were 27.9 (0.28), 27.1 (0.27) and 26.6 (0.24) hours, respectively], we found a statistically significant difference between the dorsolateral and ventral SEZ [24.7 (0.31) and 28.1 (0.35) hours, respectively] at the intermediate level (Figure 6B). Dorsolateral and ventral SEZ displayed different kinetic profiles that ultimately lead to differences in the cell cycle lengths. A significant difference in the GF was observed between the dorsolateral SEZ and the ventral SEZ [0.79 (0.03) and 0.68 (0.03), respectively].

### Analysis of Proliferating Cells Surrounding the SEZ

**Figure 7:**
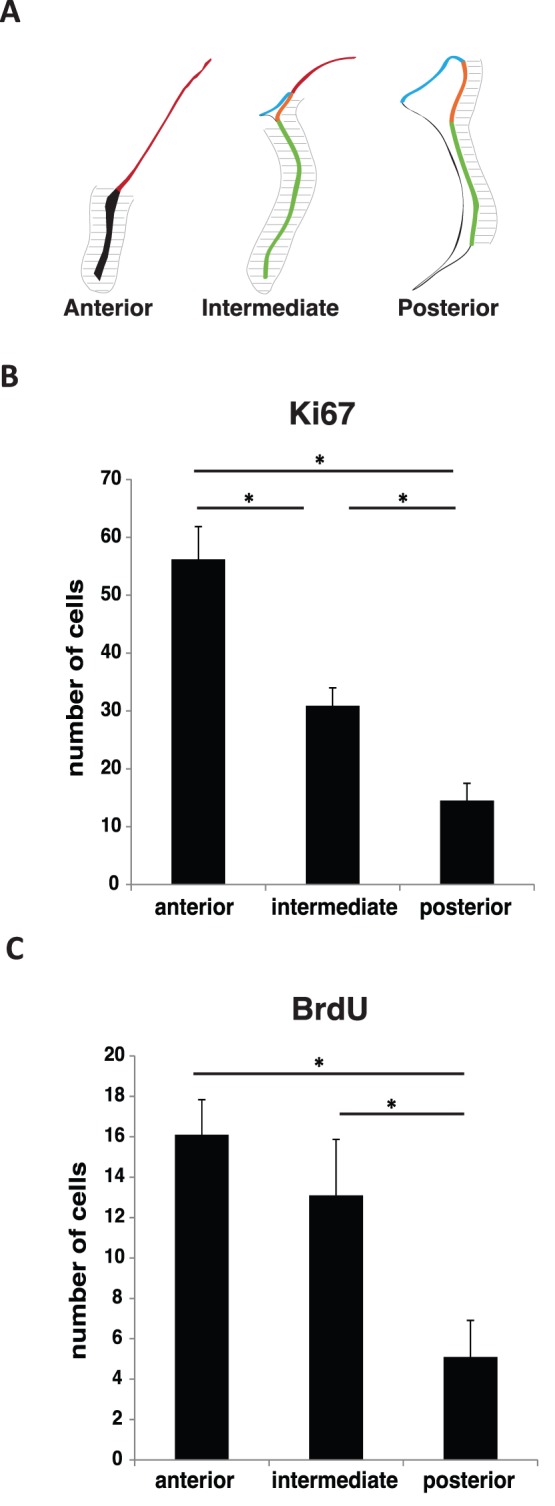
The number of cells proliferating in the vicinity of the subependymal zone decrease along the anterior-posterior axis. (A) An area within a distance of 100 µm apart from the SEZ was defined in the anterior, intermediate and posterior divisions. Ki67 (B) and BrdU (C) positive cells located in the area surrounding the SEZ, as illustrated in (A), were counted. Results are represented as number of Ki67 or BrdU positive cells per section. The threshold value for statistical significance was set at 0.05 (* p<0.05).

We were also interested in studying the number of cells proliferating in the vicinity of the SEZ; that is, within 100 µm apart from SEZ (Figure 7A), along the anterior-posterior axis. Data analysis indicates that the number of Ki67 proliferating cells in the SEZ vicinity decreased from anterior to posterior divisions (Figure 7B). These results were similar when analysed by BrdU labelling. When cells were labelled with BrdU (Figure 7C), the number of dividing cells in posterior SEZ (5±2) was decreased when compared either with the anterior or the intermediate SEZ (16±2 and 13±3, respectively; p?0.05); no differences were observed between anterior and intermediate SEZ.

## Discussion

This study provides the first unbiased stereological analysis of the SEZ proliferative pattern throughout the anterior-posterior and the dorsal-ventral axes of the adult rat brain. For this purpose the SEZ was subdivided into anterior, intermediate, posterior and post-posterior divisions (in the anterior-posterior axis) and into RMS, dorsal, dorsolateral and ventral regions (in the dorsal-ventral axis). The analyses performed, taking into consideration these divisions, revealed substantial spatial variations on cell proliferation, cell population and cell-cycle length, which reinforce the need to establish clear topographical references - which we propose herein - for studies addressing cell population dynamics in the SEZ.

The SEZ cell population comprises three main types of cells: A, B and C. Type B cells, which are quiescent stem cells that give rise to type C cells (also known as transient-amplifying progenitors), the precursors of type A cells (neuroblasts) [23]. These last two cell types are mitotically active and comprise the majority of the SEZ cell population that is labelled by short-pulse BrdU and Ki67. Evaluation of proliferation by these markers revealed heterogeneity in cell proliferation rates in the SEZ along the dorsal-ventral and anterior-posterior axes position. Specifically, with respect to the dorsal-ventral axis, the dorsolateral SEZ displayed substantially higher proliferative rates than the ventral SEZ. In the anterior-posterior axis, the anterior SEZ exhibited the highest number of proliferating cells. Of notice, the most anterior part of the SEZ comprehends a large extension of the beginning of the RMS, classically recognized as the pathway for SEZ born neuroblasts migrating towards the olfactory bulbs [3]. The fact that neuronal precursors are converging anteriorly to this pathway prompted us to investigate the contribution of the population of neuroblasts to the increased rates of proliferation in the anterior SEZ. Neuroblasts are known to migrate in response to insult/modulation [24]. Surprisingly, no differences were found in the neuroblasts population, as assessed by the number of DCX positive cells per mm^2^, at the anterior, intermediate and posterior SEZ. Conversely, at the dorsal-ventral axis the majority of the DCX positive cells were found at the dorsolateral SEZ, as observed in the DCX wholemount staining and estimated by the rates of DCX positive cells in the dorsolateral and ventral SEZ. Accordingly, the rates of proliferating neuroblasts were also reduced in ventral SEZ when compared to the dorsolateral SEZ, which is in agreement with the proliferative pattern observed herein.

As the rates of neuroblast progenitors are variable in the dorsal-ventral axis, we next asked if the stem cells from which they are derived were also differently distributed through this axis. For that purpose quiescent cells were labelled by a daily injection of BrdU over 2 weeks followed by 2 more weeks of chase to allow progenitor cells to leave the SEZ and/or dilute BrdU label. Because this method is not specific to label NSCs we further performed double staining for BrdU and GFAP, a consensual marker of NSCs [25], [26]. While this approach may label astrocytes in the proliferating niche, it is unlikely that this is a major confounder since astrocytes are not described to proliferate significantly under physiological conditions [27]. Our results show a higher rate of NSCs at the dorsolateral SEZ. This finding suggests that the number of NSCs declines from dorsal to ventral regions, which is also indicative of fewer progenitors and, thus, less proliferation. Our results are in agreement with a study that described a higher frequency of pinwheels (another method to label type B stem cells) [28] at the most dorsal part of the lateral wall, which corresponds to the herein designated dorsolateral SEZ.

Interestingly, we observed highly divergent proliferation rates along the dorsal-ventral axis. Dorsal SEZ exhibited the lowest proliferation rate of all four regions. In contrast, the dorsolateral region of the SEZ displayed the highest proliferative rate and BrdU/Ki67 ratio when compared tothe RMS, dorsal, and ventral SEZ (at intermediate levels) suggesting faster cell cycles in this region. Accordingly, the cell cycle length for DCX positive cells of the dorsolateral SEZ was confirmed to be shorter than that of the ventral SEZ. Furthermore, the rate for proliferating neuroblasts (GF) at the ventral SEZ was considerably lower than at the dorsolateral SEZ. These results reinforce the dissimilarities between the neuroblasts populations at the lateral wall.

The mitotic rates were also determined for the anterior-posterior axis. Intriguingly, the BrdU/Ki67 ratio is augmented at the posterior and post-posterior SEZ, suggesting that the cell cycle length is shortened in the most posterior portions of the SEZ, even though the proliferation rate is inferior or equivalent to that in the anterior and in the intermediate SEZ, respectively. Also, the DCX positive cell cycle lengths were not statistically significant different at the anterior, intermediate and posterior SEZ. Most likely the TAPs are also contributing to the observed BrdU/Ki67 ratio**,** even thought there was a trend in the neuroblast population to shorten the cell cycle length at more posterior levels. Although differences in NSCs proliferation along the anterior RMS have been shown (stem cells derived from distal rostral extensions of the SEZ, i.e., near the olfactory bulbs proliferate significantly more slowly than caudally placed RMS cells) [9], the same has never been shown for the SEZ.

Notably, an *in vitro* study showed that the number of label-retaining cells (commonly used to identify putative stem cells in the adult brain) obtained from 400 µm thick slices declines in posterior regions [14]. Similarly, higher frequency of pinwheels is found at the more anterior levels of the SEZ [28]. All together these observations suggest that the increased rates of proliferation at anterior levels may result from an increase in the NSCs population.

To the best of our knowledge this is the first study reporting distinct gradients in cell proliferation along the dorsal-ventral axis of the rat SEZ; it is interesting to note that it recapitulates the domains containing different types of progenitors in the germinal zone [15]. Moreover, we have estimated for the first time the cell cycle length for the neuroblasts, which is approximately 27 hours. The cell cycle length for the entire SEZ population has been estimated to be approximately 19 hours [29], [30]. Of interest, this discrepancy in time is certainly a consequence of the heterogeneity in the populations that constitute the SEZ [31], as highlighted here. In addition, it further suggests that the neuroblasts display longer cell cycles than TAPs. In fact, a short pulse BrdU labels approximately only 35% of DCX positive cells; the remaining 65% are other cellular types, mostly TAPs. Our data provides indication that the TAPs display the shorter cell cycle length of the SEZ population.

The novel methodological approach we propose here to characterize the SEZ cell population dynamics allowed a combined proliferation analysis along the anterior-posterior and dorsal-ventral axes. This approach highlighted the variations in proliferation along SEZ axes as well as the individual specificities of each dorsal-ventral region in the context of the overall SEZ proliferative rates at anterior-posterior divisions. For instance, the RMS proliferative pattern is not uniform along the SEZ, diminishing from the anterior to the posterior coordinates. On the other hand, dorsal SEZ rates of proliferating cells are higher in the posterior SEZ.

The present observations support the view that the SEZ stem cell niche is more than the initially thought thin layer of cells lining the anterior wall of the lateral brain ventricles. Besides this well-defined niche, the most ventral portion of the lateral wall [18], the RMS [9], the dorsal and the entire lateral wall of the lateral ventricles [8], [18], display progenitor cells that ultimately generate new neurons. Most importantly, it confirms dissimilarities between adult NSCs along the anatomical axes [15], [18], [31], [32]; as an example, it was demonstrated that different olfactory bulb interneurons are derived from specific locations in the SEZ [17]. As a consequence, we propose the existence of a spatial code of SEZ progenitors. This spatial code matches the regional proliferation pattern we found along the dorsal-ventral axis, supporting the concept that the spatial regionalization observed in the adult SEZ partially relates to its embryonic origin and to the distinct transcription factor expression profiles throughout the SEZ dorsal-ventral axis [15].

Adult NSCs scattered throughout the SEZ give rise to neuroblasts that converge into the RMS and migrate tangentially to the olfactory bulb [3]. However, numerous studies report the occurrence of non-tangential migration of SEZ born cells in non-physiological conditions [33]. We show here that, even in physiological conditions, there are cells proliferating in the vicinity of the SEZ that may derive from the SEZ niche. Our data demonstrate that the number of these proliferating cells under basal conditions increases towards the anterior SEZ in the same manner as SEZ proliferation. Although the fate of these proliferating cells remains to be elucidated, it is known that they increase in response to brain insults, as many SEZ derived neuronal progenitors leave the SEZ and migrate towards areas of damage [33], [34]. Assuming that some of these proliferating cells are SEZ born, we here describe a standardized method to assess non-tangential migration that should be considered in studies comprising the migration of cells outside the SEZ, in both physiological and pathological conditions.

In conclusion, this study indicates that the prevalent analysis of lateral wall of the lateral brain ventricles [35]–[38] as a proxy of the entire SEZ is biased and lacks precision as it overshadows highly relevant SEZ region specific differences. As these regional differences might also translate functional implications, their knowledge is of relevance to the development of regenerative strategies conveying the usage of endogenous SEZ cells. Thus we propose herein a SEZ topographical division model (Figure 1) that takes into consideration regional differences along the SEZ axes that will be useful to normalize and compare the results on various experimental models that assess SEZ cell dynamics.

## Materials and Methods

### Ethics Statement

This study was approved by the Portuguese national authority for animal experimentation, Direcção Geral de Veterinária (ID: DGV9457). Animals were kept and handled in accordance with the guidelines for the care and handling of laboratory animals in the Directive 2010/63/EU of the European Parliament and of the Council.

### Animals

All experiments were conducted in 10-week-old male Wistar rats (Charles River, Barcelona, Spain). Animals were maintained in 12 hours light/dark cycles at 22 to 24°C and 55% humidity and fed with regular rodent’s chow and tap water *ad libitum*. To reduce stress-induced changes in the hypothalamus–pituitary axis associated with the injection, all animals were daily handled for 1 week until the day of sacrifice.

### Administration of 5-bromo-2′-deoxyuridine (BrdU) for Proliferation Assessment and for BrdU Label Retaining Cells Estimation

For the purpose of SEZ proliferation assessment 5 animals were administered with BrdU (50 mg/Kg) intraperitoneally (*ip*) and sacrificed 2 hours later. This protocol labels SEZ fast dividing cells.

To label a quiescent pool of cells at the SEZ a group of 4 animals were daily injected with BrdU (50 mg/Kg) *ip* for 2 weeks followed by another 2 weeks period of chase. The progeny of stem cells that exit the cell cycle and retain the BrdU labelling exit the SEZ during the chase period.

### Cumulative BrdU Labelling for Cell Cycle Length Analysis

To estimate the cell cycle length of the SEZ neuroblasts population a protocol based on that previously established by Nowakowski et al [39] was performed. In accordance, three assumptions were made: 1) the proliferating population is part of a single asynchronous population 2) it is growing at a steady state and 3) there are not non-proliferating cells to consider. Based on these assumptions different groups of rats were progressively exposed to a series of BrdU injections. A total of 40 animals (n = 4 in each goup) were injected with BrdU (50 mg/Kg) *ip* at 2 hours intervals (up to a maximum of 10 time points), in a total period of 18 hours. The last BrdU injection was followed by a 0.5 hour delay before sacrifice, which allowed unlabelled proliferating cells to enter the S phase and incorporate BrdU. Thus, the first group, time point 0.5 hour, had a single BrdU injection, whereas the last group, time point 18.5 hours, received ten BrdU injections.

The interval between BrdU injections has to be shorter than the time of the S phase (Ts) to ensure that every cell that passes through the S phase incorporates BrdU at least once. This cumulative BrdU labeling will ultimately lead to saturation on the BrdU labeling of the proliferative population. At this stage every proliferating cell has incorporated BrdU and therefore a plateau is reached. At the end of the analysis a graph is obtained where the time points of BrdU injections are plotted against the percentage of the total population that is proliferating at each time point. When this percentage is constant (the plateau), it is named Growth Fraction (GF). A least squares approach was performed by using the segmental linear regression data fit. The parameters taken from the graph were used to calculate the cell cycle length (Tc). Tc was calculated from the equation slope = GF/Tc [39], [40] where GF is the growth fraction and the slope corresponds to the slope of the first linear segment. This procedure was performed for each of the SEZ regions determined in this study.

### Immunohistochemistry

Animals were anesthetized with sodium pentobarbital and transcardially perfused with cold saline for the stereological analysis of the SEZ and with 4% paraformaldehyde (PFA) in 0.01 M PBS for fluorescence immunohistochemistry. Brains were removed, embedded in O.C.T. compound and snap-frozen; serial coronal sections (20 µm) were cut in a cryostat and collected to slides for immunohistochemistry.

For the stereological analysis of the SEZ slides were post-fixed in 4% PFA in 0.01 M PBS for 30 min and antibodies against markers that evaluate cell proliferation were used: BrdU at a dilution of 1∶50 (Mouse Anti-Bromodeoxyurine, Clone Bu20a, DAKO, Spain) and Ki67 (an endogenous marker of cell proliferation) at a dilution of 1∶100 (Ki67 antigen, rabbit polyclonal antibody, Novocastra, UK). Primary antibodies were detected by the Ultravision Detection System (Lab Vision, Freemont, CA), and the reaction developed with 3,3'-diaminobenzidine substrate (Sigma Aldrich, St.Louis, MO, USA); sections were subsequently counterstained with hematoxylin.

Fluorescence immunohistochemistry was performed to label proliferating neuroblasts (double BrdU/DCX positive cells), neuroblasts (DCX positive cells) and neural stem cells (double BrdU/GFAP positive cells). The following antibodies against markers of SEZ populations were used: doublecortin (DCX) (rabbit polyclonal to doublecortin -neuroblast marker, Abcam, UK) at a dilution of 1∶500 and glial fibrillary acidic protein (GFAP) (polyclonal rabbit anti-GFAP, DAKO, Spain) at a dilution of 1∶100 together with BrdU (rat anti-BrdU, BU1/75 clone, Abcam) at a dilution of 1∶100. Fluorescent secondary antibodies (Invitrogen, Carlsbad, CA, USA), anti-rabbit and anti-rat were used to detect the primary antibodies at a dilution of 1∶1000. To label the nucleus, incubation with 4′,6-diamidino-2-phenylindole (DAPI; Sigma-Aldrich) at a dilution of 1∶1000 was performed. Primary and secondary antibodies were diluted in PBS-0.5%Triton/10% FBS and incubated overnight at 4°C, for the primary antibody, and 2 hours at room temperature, for the secondary antibody.

### Wholemount Staining

Wholemount staining for DCX was performed according to the technique described by Mirzadeh et at [41]. Briefly, the entire lateral wall, from rat brains perfused with cold saline, was dissected under a stereomicroscope and incubated in 4% PFA-0.5% Triton overnight at 4°C. Primary (anti-DCX 1∶250, Abcam) and secondary (1∶500, Invitrogen) antibodies were each incubated for 2 days at 4^a^C.

### Stereology

Estimation of cell density in the different regions of the SEZ was obtained using the Visiopharm Integrator system (VIS) software in an Olympus BX51 microscope (Olympus, Hamburg, Germany). Coronal sections for proliferation analysis comprised SEZ between bregma coordinates 2.28 mm and −3.60 mm [42].

Proliferation in the SEZ was assessed by Ki67, an endogenous marker expressed during all phases of mitosis, except for the resting phase G0 [43]; and by the exogenous marker BrdU, a thymidine analogue that is incorporated in the DNA during the S phase. The number of Ki67 and BrdU positive cells was counted and results expressed as Ki67 or BrdU positive cells per area (in mm^2^). Every sixteenth section from the anterior SEZ, bregma 2.28 mm (at this level the initial section was randomly selected to certify unbiased sampling), until posterior SEZ, bregma −3.60 mm, was analysed. The use of the VIS Software allowed delimitation, at low magnification (40×), of the areas of interest in the SEZ and the counting of Ki67 or BrdU positive cells within the defined areas at high magnification (400×). The divisions of the SEZ in the anterior-posterior axis were defined between bregma coordinates 2.28 mm and −3.60 mm (Figure 1, upper panel). Table 1 summarizes the anatomical criteria used to define anterior, intermediate, posterior and post-posterior SEZ. The SEZ anterior division starts at the beginning of the genu of the corpus callosum where a very well defined ependymal layer is observed and finishes at the end of the genu of the corpus callosum (bregma 2.28 mm to 1.44 mm); intermediate SEZ begins with the end of the genu of the corpus callosum and extends up to the decussation of the anterior commissure (bregma 1.44 mm to 0.12 mm); the posterior division of the SEZ begins at the decussation of the anterior commissure and extends to the beginning of the hippocampus, bregma −1.72 mm; the post-posterior division extends up to bregma −3.60 mm. From this position on, sparse proliferating cells were detected in the SEZ.

**Table 1:** Anterior-posterior axis landmarks of the SEZ divisions.

SEZ	Bregma coordinates (mm)	Anatomical references
**Anterior**	[2.28; 1.44[	From the beginning to the end of the genu of the corpus callosum
**Intermediate**	[1.44; −0.12[	From the end of the genu of the corpus callosum to the decussation of the anterior commissure
**Posterior**	[−0.12; −1.72[	From the decussation of the anterior commissure to the beginning of the hippocampus
**Post Posterior**	[−1.70; −3.60]	From the beginning of the hippocampus to the fusion of the dorsal and ventral parts of the lateral ventricle

Bregma coordinates are according to Paxinos & Watson (2004) [42].

Dorsal-ventral axis regionalization was performed as follows (see also Figure 1, middle panel). The dorsal SEZ located in the upper part of the lateral ventricles and the beginning of the RMS at the dorsal corner of the lateral wall. For the ventricles’ lateral wall of the SEZ the analysis was extended by evaluating for the presence of a gradient in the proliferation rate along the dorsal to ventral extension of this region. Specifically, the lateral wall of the SEZ was subdivided into contiguous 150 µm-long fragments at its length (coloured tiled map on the right of Figure 1 lower panel). The proliferation for each fragment was determined and plotted according to the dorsal-ventral axis position. This analysis comprised the intermediate and posterior SEZ and it further allowed for the division of the lateral wall into the ventral and the dorsolateral SEZ regions, illustrated in Figure 1 (middle and lower panels).

### Confocal Imaging and Quantitative Analysis

To estimate the number of neuroblasts (DCX positive cells) and proliferating neuroblasts (double DCX/BrdU positive cells) from the cumulative BrdU labelling along the anterior-posterior and dorsal-ventral axes, 6 sections per animal (2 sections at anterior levels, 2 at intermediate levels and 2 at posterior levels) were analysed. For each section, pictures were taken for the entire lateral wall of the SEZ using a confocal microscope (FV1000; Olympus) and the total number of DCX positive cells and the number of double DCX/BrdU positive cells was counted. The percentage of proliferating neuroblasts was calculated using the ratio double DCX/BrdU positive cells/total DCX positive cells.

For the single pulse BrdU labelling, double DCX/BrdU positive cells through the lateral wall, i.e., at dorsolateral and ventral regions, were counted. The rate for proliferating neuroblasts was estimated by dividing the number of double positive cells for the corresponding area. The areas were determined using the Image J software.

To estimate the total number of DCX positive cells throughout the SEZ, the same procedure as described above was performed. The DCX rates were estimated by dividing the number of double positive cells by the corresponding area.

For the BrdU label retaining cells, the rates for double GFAP/BrdU positive cells throughout the lateral wall, i.e., at dorsolateral and ventral regions, were estimated as described above for the single pulse BrdU labelling (double DCX/BrdU positive cells). The number of double GFAP/BrdU positive cells was divided for the corresponding area.

Confocal images of wholemount preparations of the lateral wall were taken with a 10× objective.

### Statistical Analysis

Data [presented as the mean (± SEM) or time (SE)] was analysed with GraphPad PRISM 5 software (GraphPad Software Inc., San Diego, CA). The analysis consisted of one-way analysis of variance (ANOVA) with Bonferroni multiple comparison post-test analysis for single-factor multiple group comparisons to determine differences between three or more groups or Student’s t test for two-group comparisons. To compare Tc between different regions a Z statistic test [40] was used. The threshold value for statistical significance was set at p<0.05 and Z>1.96.
